# Widespread local chronic stressors in Caribbean coastal habitats

**DOI:** 10.1371/journal.pone.0188564

**Published:** 2017-12-20

**Authors:** Iliana Chollett, Rachel Collin, Carolina Bastidas, Aldo Cróquer, Peter M. H. Gayle, Eric Jordán-Dahlgren, Karen Koltes, Hazel Oxenford, Alberto Rodriguez-Ramirez, Ernesto Weil, Jahson Alemu, David Bone, Kenneth C. Buchan, Marcia Creary Ford, Edgar Escalante-Mancera, Jaime Garzón-Ferreira, Hector M. Guzmán, Björn Kjerfve, Eduardo Klein, Croy McCoy, Arthur C. Potts, Francisco Ruíz-Rentería, Struan R. Smith, John Tschirky, Jorge Cortés

**Affiliations:** 1 Smithsonian Marine Station, Smithsonian Institution, Fort Pierce, Florida, United States of America; 2 Smithsonian Tropical Research Institute, Smithsonian Institution, Panama City, Panama; 3 Departamento de Biología de Organismos, Universidad Simón Bolívar, Caracas, Venezuela; 4 Massachusetts Institute of Technology, Sea Grant Program, Cambridge, Massachusetts, United States of America; 5 Departamento de Estudios Ambientales, Universidad Simón Bolívar, Caracas, Venezuela; 6 Discovery Bay Marine Laboratory, Centre for Marine Sciences, University of the West Indies, St. Ann, Jamaica; 7 Instituto de Ciencias del Mar y Limnología, Universidad Nacional Autónoma de Mexico, Puerto Morelos, Mexico; 8 Office of Insular Affairs, US Department of the Interior, Washington DC, United States of America; 9 Centre for Resource Management and Environmental Studies, University of the West Indies, Cave Hill, Barbados; 10 Global Change Institute, The University of Queensland, Brisbane, Queensland, Australia; 11 University of Puerto Rico, Mayagüez, Puerto Rico; 12 University of the West Indies, Port of Spain, Trinidad and Tobago; 13 Instituto de Tecnología y Ciencias Marinas, Universidad Simón Bolívar, Caracas, Venezuela; 14 Environment and Economy Directorate, Dorset County Council, Dorchester, Dorset, United Kingdom; 15 Centre for Marine Sciences, University of West Indies, St. Ann, Jamaica; 16 Brewster Academy, Wolfeboro, New Hampshire, United States of America; 17 American University of Sharjah, Sharja, United Arab Emirates; 18 Department of Environment, Cayman Islands Government, Georgetown, Grand Cayman; 19 School of Ocean Sciences, Bangor University, Gwyneth, United Kingdom; 20 University of Trinidad and Tobago, Chaguaramas, Trinidad and Tobago; 21 Bermuda Aquarium Museum and Zoo, Flatt’s, Bermuda; 22 American Bird Conservancy, International Program, Washington DC, United States of America; 23 Centro de Investigación en Ciencias del Mar y Limnología, Universidad de Costa Rica, San José, Costa Rica; Department of Agriculture and Water Resources, AUSTRALIA

## Abstract

Coastal ecosystems and the livelihoods they support are threatened by stressors acting at global and local scales. Here we used the data produced by the Caribbean Coastal Marine Productivity program (CARICOMP), the longest, largest monitoring program in the wider Caribbean, to evidence local-scale (decreases in water quality) and global-scale (increases in temperature) stressors across the basin. Trend analyses showed that visibility decreased at 42% of the stations, indicating that local-scale chronic stressors are widespread. On the other hand, only 18% of the stations showed increases in water temperature that would be expected from global warming, partially reflecting the limits in detecting trends due to inherent natural variability of temperature data. Decreases in visibility were associated with increased human density. However, this link can be decoupled by environmental factors, with conditions that increase the flush of water, dampening the effects of human influence. Besides documenting environmental stressors throughout the basin, our results can be used to inform future monitoring programs, if the desire is to identify stations that provide early warning signals of anthropogenic impacts. All CARICOMP environmental data are now available, providing an invaluable baseline that can be used to strengthen research, conservation, and management of coastal ecosystems in the Caribbean basin.

## Introduction

Changes at local and global scales are influencing our oceans, altering their health and the benefits we receive from them. Here we use the terms global and local to define scales of action of anthropogenic stressors, ranging from disturbances acting on broad spatial scales, such as ocean warming, to those acting at very localized scales, such as dredging [1,2]. These changes have affected the health of marine ecosystems and the services they provide [3] and may threaten coastal livelihoods and food security [4]. Long-term measurements of environmental parameters over wide geographic regions are necessary to understand the rate of change at global and local scales. Such a strategy provides information that informs identification of threatened areas and provides potential explanations for and predictions of ecosystem responses. A long-term approach also allows the assessment of progress towards management objectives and planning for mitigation or adaptation accordingly [5].

Increases in temperature and decreases in water quality are common indicators of changes in the oceans at global and local scales, respectively [1,6]. Increases in greenhouse gases released by human activities have altered ocean temperature, generally by warming [7]. In the Caribbean, analyses of remote sensing data indicate that most areas have warmed at rates that range from 0.2 to 0.5°C dec^-1^ during the last three decades [8]. These increases in temperature have been positively correlated with increases in the frequency and prevalence of coral bleaching and, in some cases, diseases affecting coral reef species across the region [9–11]. The localized influence of human stressors, on the other hand, has been manifested as decreases in water quality driven by increased pollution resulting from rapid development and habitat conversion [1]. Decreases in water quality have also been mapped using satellite information, but only at regional scales, showing increases in turbidity in several localized areas in the Caribbean (e.g. [12,13]).

Optical remote sensing has been a pivotal tool in quantifying changes in the oceans at global and regional scales [14], however, this tool is not well suited to study patterns and processes at the land-sea interface [15]. While this technology can sample the globe cheaply and repeatedly over a large area, it can be inaccurate in coastal areas. The inaccuracy of optical remotely-sensed data close to the coast is related to two main issues: high cloud coverage in coastal areas that blocks the view from satellites, and the presence of land that contaminates the signal received by the sensor [15,16]. Additionally, the complex optical signal of coastal waters hinders the quantification of water quality along the coast; the complex mixture of components in coastal waters makes the quantification of the separate constituents very difficult, and shallow bottoms can look very similar to heavily turbid regions. As a result, water quality can be measured using remote sensing only in particular locations using algorithms that are heavily reliant on *in situ* data [15,16]. Thus *in situ* measurements from monitoring programs may play an important role in quantifying patterns in coastal areas.

Long-term *in situ* datasets documenting temporal changes in the environment of coastal areas, where most economically valuable ecosystems are located, are limited [17,18]. Most *in situ* datasets that record ocean conditions focus on open-ocean areas (e.g. SeaBASS: [19]), and do not provide repeated measurements that allow for the quantification of changes at fine spatial scales (e.g. the World Ocean Database: [20]). First of its kind in the wider Caribbean, the international Caribbean Coastal Marine Productivity program (CARICOMP) was established almost 30 years ago to fill this gap [21]. The CARICOMP long-term program was developed to study processes at the land-sea interface and understand productivity, structure and function of the three main coastal habitats (mangroves, seagrass meadows, and coral reefs) across the region [21,22]. Together with biological monitoring, the CARICOMP network has collected environmental data since 1992 using simple, standardized methods [21–23].

Here we used the environmental data collected by CARICOMP’s monitoring network to quantify long-term changes in oceanographic conditions in coastal habitats in the wider Caribbean. We focused our analyses on temperature and visibility, two proxies of global and local chronic stressors in marine environments. We had two aims. First, quantifying significant changes in these environmental variables over time. Second, understanding if these stressors are influencing the entire basin in a homogeneous way, and if not, what factors (i.e. water movement, rainfall, and human influence) could explain differences among sites. In this study we not only synthesize the information in this unparalleled dataset (which is made available with this publication), but provide guidelines for the better selection of monitoring sites if future aims include identifying early warning signals of change.

## Materials and methods

### CARICOMP dataset

Beginning in 1992, CARICOMP established permanent monitoring stations in mangrove, seagrass, and coral reef habitats. Effort was made to select stations that specifically avoided anthropogenic sources of disturbance, particularly coastal development and pollution [21]. Weekly (whenever possible) physical measurements were taken at each station between 10:00 and 12:00 local standard time. Measurements consisted of water temperature (°C), salinity, and visibility (m). Temperature and salinity were measured with a field thermometer and a refractometer at 0.5 m depth at all habitats. Visibility was measured with a Secchi disk in seagrass (measured horizontally 0.5 m below the surface, as these habitats are often too shallow for a standard vertical measurement) and reef habitats (typically measured vertically over the drop-off), and can be assumed to indicate water quality at the surface. Secchi depth is strongly correlated to the amount of particulate material in the water column and it has been used as a cheap, fast, and simple proxy for visibility and water quality [24]. We are aware, however, that this is only one of the multiple environmental variables that characterize water quality at a site, and that a full assessment of this component would require the measurement of other variables (e.g. concentration of nutrients, pollutants, dissolved matter).

Data from previously published CARICOMP databases and updates provided directly from individual researchers at CARICOMP stations were compiled into a uniform format. All environmental CARICOMP data are available in the Supporting Information (a description of all stations is in Tables A and B in S1 File and the data are in S2 File). Although information from all three variables is included in the appendix, to address the aims of this research only temperature and Secchi data were analyzed.

Simple mixed effect models for the assessment of differences among habitats (fixed factor) including all stations (as random factor) were fitted with the R package *lmerTest* [25], which provides additional F statistics and p-values for factors calculated based on Satterthwaite’s approximations. Satterthwaite’s method allows for the calculation of the denominator degrees of freedom as a function of the variance of the parameter estimate [26], therefore estimating significance in mixed effect models which is generally problematic [27].

**Fig 1:**
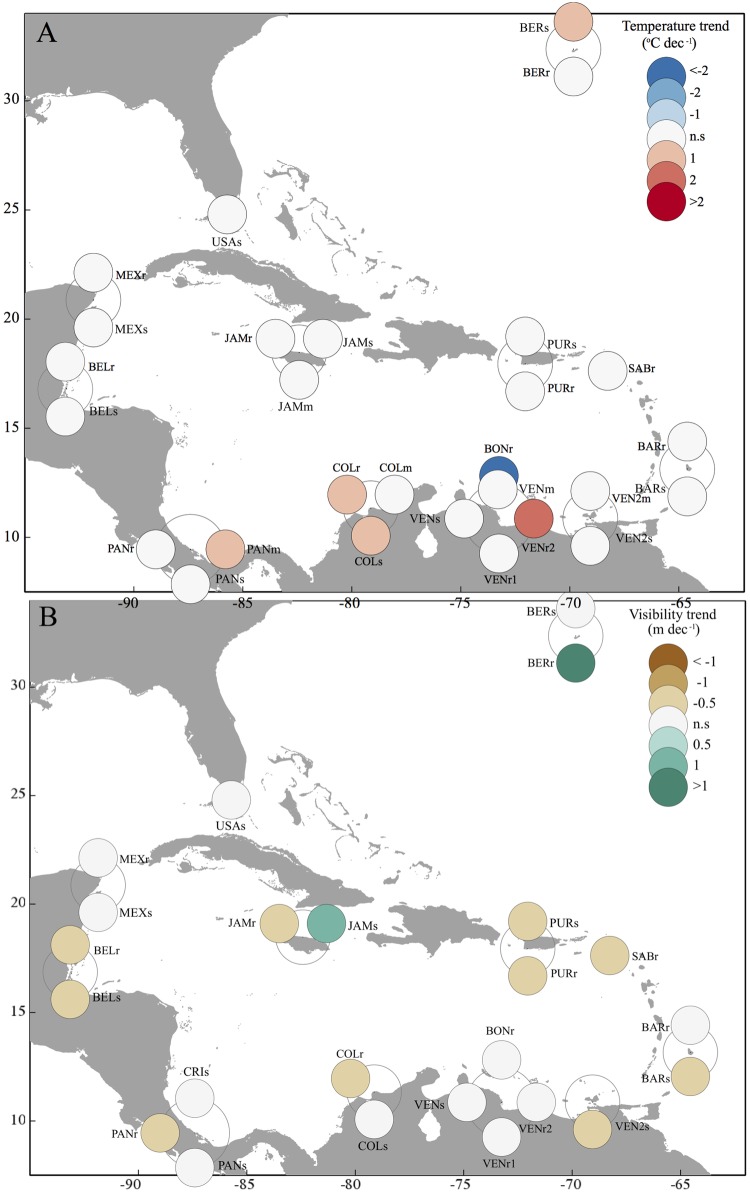
Changes in temperature and visibility throughout the CARICOMP network. Map of CARICOMP stations showing significant increases, decreases, or non-significant trends for temperature (A) and visibility (B). Labels as in Table 1, with upper case letters indicating the location and lower case the habitat.

Monthly averages were calculated from the weekly data for each station. To ensure meaningful quantification of a linear trend, only stations with data for at least three years and a minimum of 30 monthly records were included in subsequent analyses (60% of the sites: Table 1, Fig 1).

**Table 1:** Description of sites. CARICOMP stations with long-term data (at least three years and 30 monthly records).

Country	Site	Habitat	Station acronym	Latitude	Longitude	Year range
Barbados	Bellairs	Coral Reef	BARr	13.192	-59.642	11/1992-12/1999
Barbados	Bellairs	Seagrass Beds	BARs	13.068	-59.578	11/1992-09/1996
Belize	Carrie Bow Cay	Coral Reef	BELr	16.800	-88.067	01-1993/07-2015
Belize	Carrie Bow Cay	Seagrass Beds	BELs	16.825	-88.099	01-1993/07-2015
Bermuda	Hog Breaker Reef	Coral Reef	BERr	32.344	-64.865	09-1992/12-2002
Bermuda	North Seagrass	Seagrass Beds	BERs	32.401	-64.799	09-1992/12-2002
Bonaire, N.A.	Barcadera Reef	Coral Reef	BONr	12.195	-68.301	08/1994-12/1997
Colombia	Chengue Bay	Coral Reef	COLr	11.328	-74.128	09-1992/06-2011
Colombia	Chengue Bay	Mangrove	COLm	11.317	-74.128	09-1992/06-2011
Colombia	Chengue Bay	Seagrass Beds	COLs	11.321	-74.127	09-1992/06-2011
Costa Rica	Rio Perezoso	Seagrass Beds	CRIs	9.737	-82.807	03-1999/05-2015
Jamaica	Discovery Bay	Coral Reef	JAMr	18.472	-77.414	09-1992/02-2002
Jamaica	Discovery Bay	Mangrove	JAMm	18.469	-77.415	09-1992/02-2002
Jamaica	Discovery Bay	Seagrass Beds	JAMs	18.471	-77.414	09-1992/02-2002
Mexico	Puerto Morelos	Coral Reef	MEXr	20.878	-86.845	10-1992/10-2005
Mexico	Puerto Morelos	Seagrass Beds	MEXs	20.868	-86.867	09-1992/10-2005
Panama	STRI_colo	Coral Reef	PANr	9.349	-82.266	06-1999/05-2015
Panama	STRI_colo	Mangrove	PANm	9.352	-82.259	02-1999/05-2015
Panama	STRI_colo	Seagrass Beds	PANs	9.352	-82.258	06-1999/05-2015
Puerto Rico	La Parguera	Coral Reef	PURr	17.935	-67.049	01-1993/12-2014
Puerto Rico	La Parguera	Seagrass Beds	PURs	17.955	-67.043	01-1993/12-2014
Saba, N.A.	Ladder Labyrinth	Coral Reef	SABr	17.626	-63.260	09-1992/04-1997
USA	Long Key	Seagrass Beds	USAs	24.800	-80.717	07-1996/06-2004
Venezuela	P.N. Morrocoy—Caiman	Coral Reef	VENr1	10.852	-68.232	09-1992/11-1999
Venezuela	P.N. Morrocoy—Cayo Sombrero	Coral Reef	VENr2	10.881	-68.213	02-2000/11-2012
Venezuela	P.N. Morrocoy	Mangrove	VENm	10.836	-68.261	01-1993/11-2012
Venezuela	P.N. Morrocoy	Seagrass Beds	VENs	10.858	-68.291	09-1992/11-2012
Venezuela	Punta de Mangle	Mangrove	VEN2m	10.864	-64.058	01-1993/12-2003
Venezuela	Punta de Mangle	Seagrass Beds	VEN2s	10.864	-64.058	01-1993/12-2003

### Global and local-scale changes across the Caribbean

To assess global and local-scale changes across the Caribbean, we focused our analyses on changes in temperature and visibility, which as previously noted, are common proxies for change at each scale. Long-term trends and significance were calculated considering serial correlation, a characteristic of the data that, if not taken into account, violates the assumption of independence of most regression analyses and influences the magnitude and significance of trends [27].

Following Weatherhead et al. [27], for temperature (*T*), we fitted a non-linear model with the form:

$$T=\mu +S_{t}+\frac{\omega t}{12}+N_{t}$$ (1)

Where the temperature at time *t* in months is a function of a constant term *μ*, a seasonal component with sinusoidal form *S*_*t*_, a linear trend of rate °C year^-1^, and residuals *N*_*t*_. In this model, the seasonal component is allowed to include up to two cycles, and is described by the formula:

$$S_{t}\;=\;\sum_{j\;=\;1}^{4}\beta_{1,j}sin\frac{2\pi jt}{12}+\beta_{2,j}cos\frac{2\pi jt}{12}$$ (2)

Where *t* is the number of months, and *β* are parameters to be estimated. And the residuals have an AR-1 autocorrelation form, the simplest form of autocorrelation (i.e., the similarity between a time series and a lagged version of itself). That is, the residuals at time *t* are a function of the residuals at time *t-1* (i.e. the temporal “memory” of the time series has a one month lag), depending on the station-specific autocorrelation parameter *νΦ*, along with the noise (*ϵ*_*t*,_ [27]):

$$N_{t}=\phi N_{t-1}+\epsilon_{t}$$ (3)

For visibility (*V*), we fitted a non-linear model that follows the approach described above but without the seasonal component:

$$V=\mu +\frac{\omega t}{12}+N_{t}$$ (4)

In this model, *V* at a given time *t* in months is a function of a constant term *μ*, a linear trend of rate m year^-1^, and residuals, *N*_*t*_ also assumed to have a AR-1 autocorrelation form (Eq 3)

The models were fitted using generalized squares and the package *nlme* in *R* [28]. Initial estimates for *μ* and were obtained through simple linear regression, and initial values of 1 were used for all *β*’s.

### Correlates of global and local-scale changes

Global and local-scale stressors can be exacerbated or dampened by local conditions related to water movement, with circumstances that increase the flush of water potentially less conducive to warming and decreases in visibility [29,30]. We examined the effects of water movement through the inclusion of two variables: wave exposure and current speed. Additionally, trends in visibility can be driven by human influence (with areas of rapid population increases expected to lose visibility), and could also be influenced by trends in rainfall (with stations that are getting wetter anticipated to show increased turbidity); therefore these two variables were also included to explain trends for this response variable. This way, we characterized each station with the explanatory variables: (1) average wave exposure; (2) average current speed; (3) changes in human population density; (4) trend in rainfall. Due to the lack of consistent *in situ* datasets for all stations, modelled or remote sensing sources were used to derive explanatory variables. Below we briefly describe each dataset.

Wind-driven wave exposure for each station is dependent on the wind patterns and the configuration of the coastline, which defines the *fetch*, or the length of water over which a given wind has blown to generate waves. To calculate wave exposure, wind speed and direction data at each location were acquired from the QuickSCAT (NASA) satellite scatterometer from 1999 to 2008 at 25 km spatial resolution [31]. Coastline data were obtained from the Global Self-consistent, Hierarchical, High-resolution, Shoreline (GSHHS v 2.2) database which provides global coastline at 1:250,000 scale [32]. From these datasets wave exposure was calculated using the methods based on wave theory described in Chollett et al. [33] for 32 fetch directions and the coastline data at full resolution. Average wave exposure at each station was calculated in *R* with the aid of the packages *maptools*, *raster*, *rgeos*, and *sp* [34–37].

Average surface current speed was extracted from the ocean model HYCOM [38]. We used global data-assimilative runs at 1/12° of spatial resolution for the period 2008–2011. The HYCOM model is forced by wind stress, wind speed, heat flux, and precipitation and the system uses *in situ* temperature and salinity profiles to improve estimates, providing the most detailed and comprehensive global dataset of ocean currents available to date [39].

Gridded human population density data for the years 1990 and 2000 (the most recent dataset available at that spatial detail) were obtained from the Global Rural-Urban Mapping Project, Version 1 (GRUMPv1: [40]). These years coincide with the period when most of the CARICOMP took place, with the time series beginning on average in February 1994 and finishing on average in September 2007 (Table 1). We used the adjusted population density grids as inputs, which provide population density in persons per square kilometre using census information but also observations of night lights to delineate the extent of urban areas. From these datasets we extracted the number of people within a buffer of 1-degree diameter around each station, and then calculated the difference in population between the years 2000 and 1990, which captures a proxy for broad impacts of human population expansion on coastal ecosystems. A one degree buffer was considered a reasonable range at which many human impacts might affect coastal ecosystems, as demonstrated in previous studies [41].

Satellite rainfall data were extracted from the GPCP v2.2 combined precipitation dataset, which merges satellite and gauge precipitation values in monthly estimates of total precipitation from 1986 to 2016 (i.e. 37 years of data) at 2.5° spatial resolution. This is the longest, most accurate global dataset of rainfall available to date [42,43]). For each station, trends were calculated from these monthly means taking into account the temporal autocorrelation of the data (Eq 4).

When trends are non-significant their value is uninformative (e.g. a trend in temperature of 2°C year^-1^ with a p value of 0.8 is meaningless), hindering the use of the actual trend values as a response variable in quantitative analyses. We therefore transformed the continuous data (i.e. trend values in temperature and visibility) into nominal data (i.e. trend categories) by classifying trends as non-significant, significantly increasing or significantly decreasing. We then used multinomial regression models to identify what factors were relevant at explaining the observed trend categories in temperature and visibility. Multinomial regression is a method used to generalize logistic regression where the response variable is nominal and has more than two classes, in which the log odds of the outcomes are modelled as a linear combination of predictor variables. Here, we modelled trends in temperature as a function of wave exposure and currents, and trends in visibility as a function of wave exposure, currents, changes in human population, and trends in rainfall. Multinomial regression was carried out using the package *nnet* in R [44]. All figures were produced using the package *ggplot2* in R [45].

## Results

### CARICOMP dataset

CARICOMP collected data at 48 stations in 18 countries/territories across the wider Caribbean (Tables A and B in S1 File). Participants in the network have sampled environmental data from 20 reefs, 19 seagrass meadows, and 9 mangrove forests since 1992. Data collection is ongoing at some stations.

**Fig 2:**
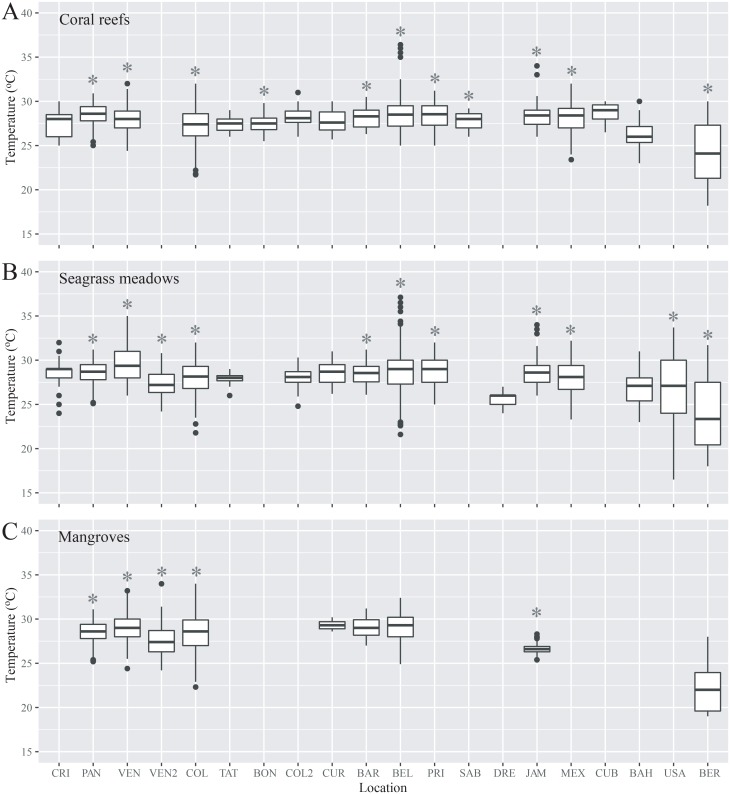
Sea temperature throughout the CARICOMP network. Sea temperature in each site and habitat in the CARICOMP network, all data are presented, including all years (i.e. since 1992) and all stations, with and without long-term (> 3 years) data: (A) coral reefs; (B) seagrass meadows; and (C) mangroves. In boxplots, lines represent means, boxes 25 and 75% quantiles, whiskers 1.5 inter-quartile ranges and dots outliers. Sites are: Costa Rica (CRI), Panama (PAN), western Venezuela (VEN), eastern Venezuela (VEN2), Colombia (COL), Trinidad y Tobago (TAT), Bonaire (BON), northern Colombia (COL2), Curaçao (CUR), Barbados (BAR), Belize (BEL), Puerto Rico (PUR), Saba (SAB), Dominican Republic (DRE), Jamaica (JAM), Mexico (MEX), Cuba (CUB), the Bahamas (BAH), United States (USA), and Bermuda (BER). Sites with an asterisk were included in subsequent analyses.

**Fig 3:**
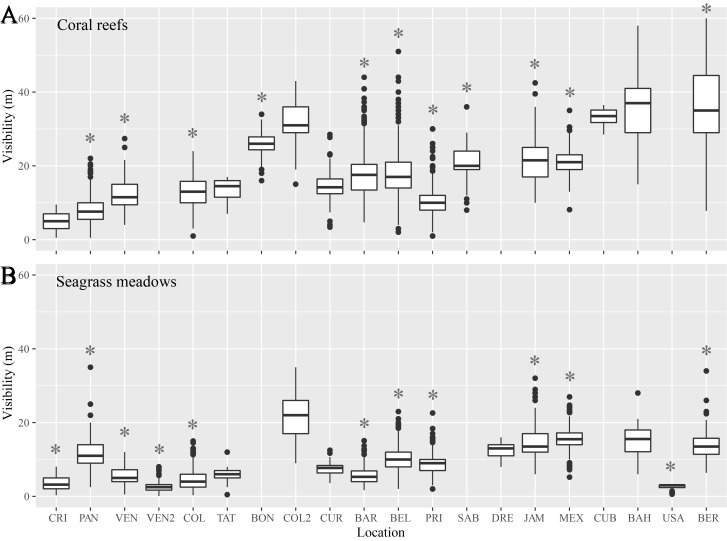
Visibility throughout the CARICOMP network. Visibility in each site and habitat in the CARICOMP network, all data are presented, including all years (i.e. since 1992) and all stations, with and without long-term (> 3 years) data: (A) Coral reefs; and (B) Seagrass meadows. In boxplots, lines represent means, boxes 25 and 75% quantiles, whiskers 1.5 inter-quartile ranges and dots outliers. Sites are: Costa Rica (CRI), Panama (PAN), western Venezuela (VEN), eastern Venezuela (VEN2), Colombia (COL), Trinidad y Tobago (TAT), Bonaire (BON), northern Colombia (COL2), Curaçao (CUR), Barbados (BAR), Belize (BEL), Puerto Rico (PUR), Saba (SAB), Dominican Republic (DRE), Jamaica (JAM), Mexico (MEX), Cuba (CUB), the Bahamas (BAH), United States (USA), and Bermuda (BER). Sites with an asterisk were included in subsequent analyses.

Water temperature and visibility were variable throughout the region (Figs 2 and 3). Average temperature ranged from about 22°C in Bermuda to almost 30°C in Cuba, but many stations showed relatively similar values (Fig 2). There were no clear differences in temperature among seagrass, mangroves, and coral reefs (mixed effect model with location as random effect, F = 0.74, p = 0.48). Visibility, only measured in reef and seagrass habitats, also showed large variability among stations, with a minimum of about 3 m at the seagrass meadow off eastern Venezuela, and a maximum of 37 m at the reef in the Bahamas (Fig 3). Locations with lower values of visibility also showed the greatest variability. As expected, there were clear differences in visibility between habitats, with higher values in coral reefs (mixed effect model with location as random effect, F = 18.22, p < 0.001). Sixty percent of the CARICOMP stations (described in Table 1) included long-term records and were therefore suitable candidates for the estimation of long-term trends in subsequent analyses.

### Global and local-scale changes across the Caribbean

**Fig 4:**
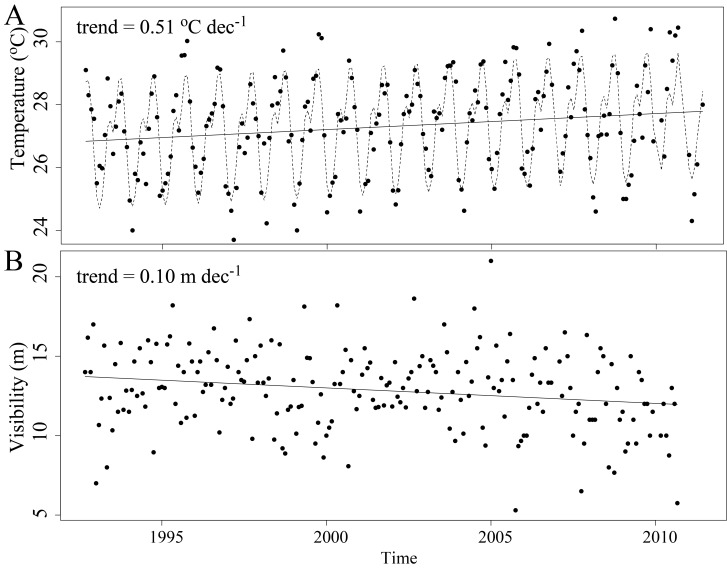
Time series example. Time series for sea temperature (A) and visibility (B) for the reef at Chengue Bay (Colombia), showing significant increases in temperature and significant decreases in visibility. For temperature, the model fit takes into account both seasonality (sinusoidal line) and a linear trend (straight line).

Data collected by the CARICOMP network offered evidence of widespread local, but not global-scale changes across the wider Caribbean using visibility and sea temperature as proxies. While a few stations showed evidence of warming, about half the stations showed evidence of decreased visibility (Fig 1). The mixed effects models represented the temporal variability in the oceanographic variables well, capturing both the seasonality (for temperature) and long-term linear trends (Fig 4, Tables A and B in S3 File).

There was large spatial variability in temperature and visibility trends across the CARICOMP network (Fig 1). Of the 28 reef, seagrass, and mangrove stations, 18% (1 mangrove, 2 seagrass meadow, and 2 coral reef stations) showed a significant increasing trend in temperature, and only one (Bonaire reef) showed a significant decrease (Fig 1A, Table A in S3 File). On the other hand, of the 24 reef and seagrass stations, 42% (4 seagrass meadows and 6 reefs) showed a significant decreasing trend in visibility, and two stations (Jamaica seagrass and Bermuda reef) showed a positive trend (Fig 1B, Table B in S3 File). Neither warming nor decreases in visibility were observed to be more common in any of the three habitats monitored (Chi-squared tests, p > 0.05).

### Correlates of global and local-scale changes

**Fig 5:**
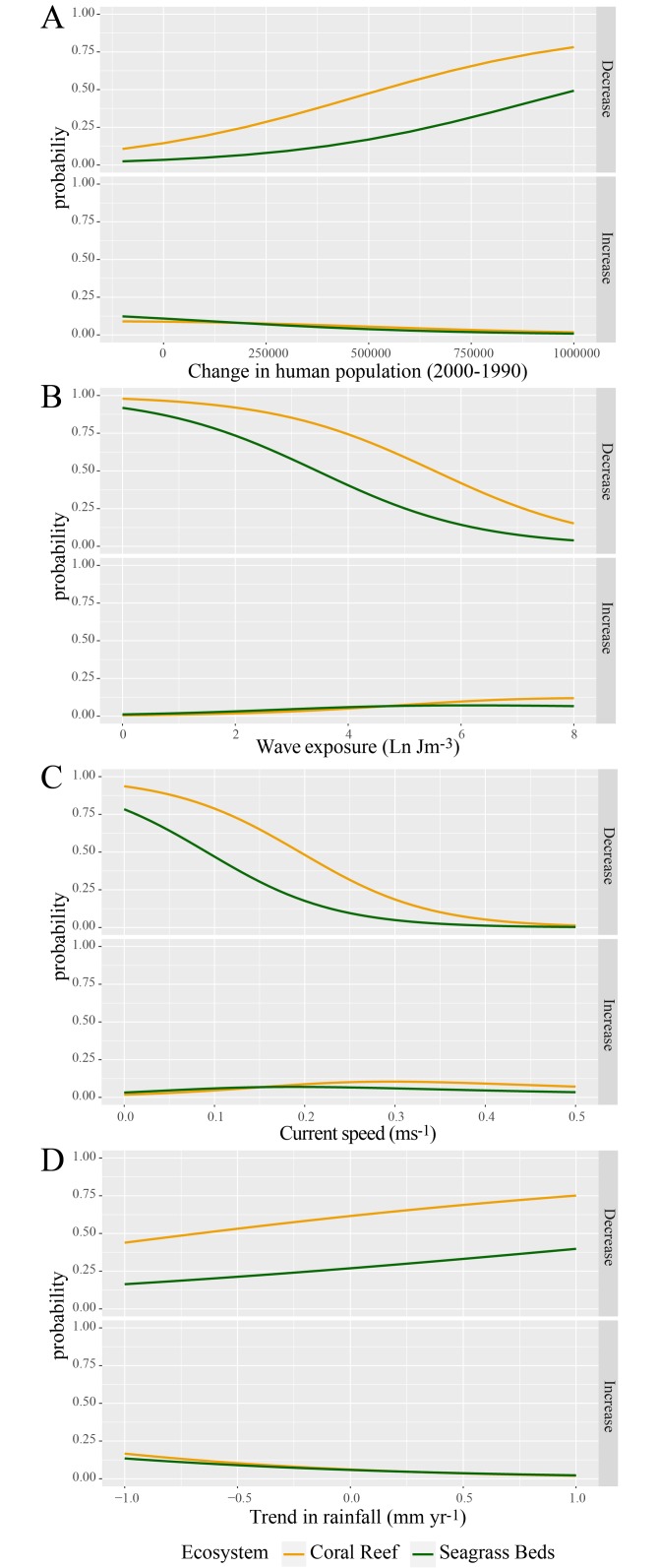
Explaining trends in visibility. Predicted probability of decreases and increases in visibility (as per right-hand labels of the top and bottom panels, respectively) against changes in human population (A), wave exposure (B), current speed (C), and trend in rainfall (D).

The presence of negative, positive, or non-significant trends in temperature was not explained by either of the two local factors assessed (wave exposure and currents, multinomial regression, p > 0.05 for both variables). Trends in visibility were explained by all variables, that is, changes in human population, wave exposure, current speed, and trend in rainfall (multinomial regression, p < 0.01). Decreases in visibility were more likely to occur in areas where human population (and associated coastal development) has increased the most (Fig 5A). Oceanographic and atmospheric variables have the ability to modulate changes in visibility (Fig 5B–5D). Long-term decreases in visibility were more likely to occur at stations with slow water motion, characterized either by low exposure (Fig 5B, top panel) or low current speed (Fig 5C, top panel). Conversely, long-term increases in visibility were more likely at stations with high wave exposure and current speed, although these variables had a very small effect in driving significant long-term increases in visibility (Fig 5B and 5C, bottom panels). Finally, decreases in visibility were also more likely to occur in areas that were getting wetter, and increases were more likely in areas that were getting drier (Fig 5D). The functional responses to these explanatory variables were similar irrespective of habitat (Fig 5).

## Discussion

The longest and most spatially comprehensive *in situ* monitoring effort in the wider Caribbean provides evidence of widespread local changes within the basin. This is a relatively unexpected result, given that CARICOMP stations were intended to be established in pristine areas under minimal local impacts that could serve as a baseline against which to measure degradation [21]. However, 15 years ago it was already suggested that some stations were being impacted by human activities [22]. Results presented here support this statement, agree with results of localized studies in some of these locations (e.g. [46–50]), and indicate that human impacts on coastal habitats are ongoing and pervasive within the Caribbean basin.

CARICOMP’s time series do not show widespread evidence of long-term warming at coastal stations in the wider Caribbean. These findings contrast with a global study that showed prevalent warming along the world’s coasts using 30 years of satellite data [30], and a regional study which showed significant warming throughout most of the Caribbean basin using 25 years of satellite temperatures [8]. The lack of signal in the CARICOMP time series can be attributed to two related issues: the larger variability of *in situ* temperature data and the need for longer time series to detect significant trends. Satellites measure temperature at the ‘skin’ of the ocean surface, which is more stable [51], and ignores subsurface temperature patterns that are more variable at multiple temporal scales (from minutes to decadal: [52]). Therefore, *in situ* temperature data are more variable making trend estimation more difficult. Low precision of *in situ* measurements due to external influences (such as changes in sampling methodology, observers, instrumentation or gaps in the time series: [27,53]) could also increase variability and limit the ability to detect trends. Besides the issue of increased variability, the inability to detect trends might be related to the length of the CARICOMP time-series (from 3 to 22 years). This timeframe may provide insufficient statistical power to assess long-term changes in temperature due to intrinsic characteristics of the location, particularly in stations where the magnitude of the trend is small, the memory (i.e. temporal autocorrelation) is high, or temperature is especially variable [27].

**Fig 6:**
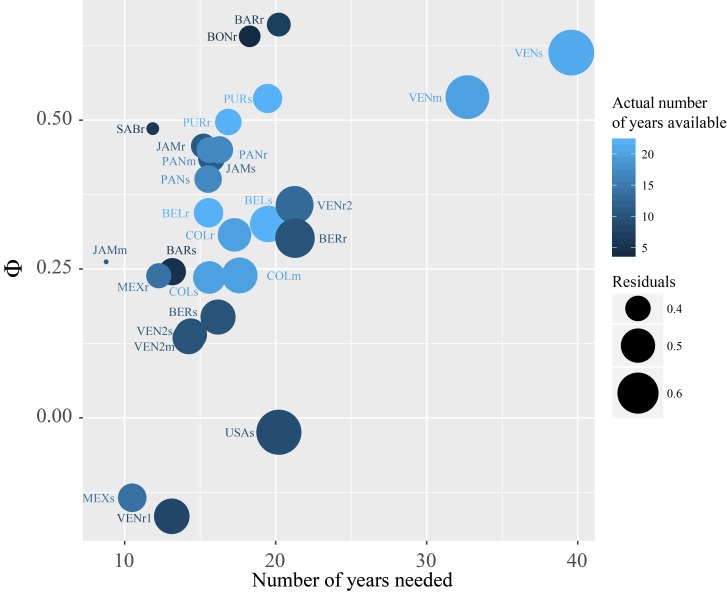
Explaining the lack of trends in temperature. Number of years needed to detect a trend in temperature of 0.05°C year^-1^ as a function of the autocorrelation of the noise (φ) and the residuals of each station [27]. Also shown in color the actual number of years of data available for each station. Note that to identify trends of different magnitudes, different number of years might be required.

Site-specific information on the inherent characteristics of the time-series can be used to aid in the identification of monitoring sites that are cost-effective in the sense that they have the power to detect trends earlier [27], if the detection of early changes is the main objective of the monitoring. Significant trends will be detected faster at sites characterized by low variability and temporal autocorrelation of the noise, which is a measure of the ‘memory’ or inertia of the time-series. For example, within the CARICOMP network, the time period to detect an expected change varies greatly among stations (Fig 6, Table A in S3 File). Within this dataset, given the variability and memory of the time-series, Puerto Morelos in Mexico would need the shortest sampling to identify changes in temperature (about ten years), and it might be a good location to identify trends in temperature early. In contrast the seagrass meadow and mangrove stations in eastern Venezuela might need the longest time series to detect a significant trend (Fig 6, Table A in S3 File). This result is not rare; research in atmospheric [27] and oceanographic [54,55] science has shown that for most expected environmental changes, several decades of high-quality data may be needed to detect significant trends. For example, many years of continuous data were needed to distinguish a climate change trend in pH and sea surface temperature (about 15 years), chlorophyll concentration and primary production (between 30 and 40 years) from the natural background variability [54,55]. The process of deciding which sites may be useful for future detection of trends is very similar to conducting power analysis to estimate the number of samples needed to detect a particular effect. This type of analysis can be done if the data has already been collected (as in the example in Fig 6) or before collecting the data, assuming a range of effect sizes, autocorrelation, and noise [27], and taking into account any external forces that might affect the accuracy of the data (see previous paragraph; [53]). This information can be used to set realistic expectations on trend detectability at different sites. It could also be used to identify sites for further monitoring of chronic impacts [56] and early detection of trends, after accounting for important factors such as the relevance of the site to answer the s scientific question at hand, or logistic factors such as accessibility and maintenance of the monitoring site.

Decreases in visibility were related to changes in human density, which increased in all but one (Bonaire) of the CARICOMP stations assessed. The effects of local anthropogenic impacts can be modulated, however, by local hydrodynamic and weather conditions. Areas with high flush of marine water and/or drier weather are less vulnerable to deteriorating visibility. Waves and currents flush sediments, nutrients, and pollutants and determine the spatial variability in visibility patterns [29, 57]. Decreased rainfall, on the other hand, diminishes runoff reaching the stations, thus improving visibility [58]. The Caribbean basin is getting drier [59] due to the intensification of the Caribbean Low Level Jet [60] and warming in the Atlantic [61]. Because rainfall is predicted to decrease further [60], we expect that rainfall and runoff will play diminished roles in exacerbating local stressors in the basin in the near future. Knowledge of the factors that modulate the detection of trends in visibility can also assist in the identification of the best monitoring sites for early warning signal detection. In this sense, sites with vigorous water movement should be avoided if the desire is the early detection of water quality degradation in coastal areas.

Chronic decreases in coastal water quality can be linked to the increase in marine diseases [62] and the demise of seagrass [63] and coral reef ecosystems [57]. Furthermore, declines in water quality have been linked to economic losses such as decreases in property value and tourism revenues (reviewed in [64]). Results presented here pinpoint areas that might require management interventions. Such interventions may include identifying the cause of decreased water quality, and implementing changes in management practices and long-term commitments towards change. Improving water quality could also have the added benefit of improving resilience of coastal ecosystems to other disturbances, such as climate change [65,66].

CARICOMP’s environmental dataset provides an invaluable baseline that can be used to strengthen research, conservation, and management of coastal ecosystems in the Caribbean basin. First, the dataset provides context for other local studies, aiding comparisons and understanding of observations at single locations [67]. CARICOMP’s environmental measurements also provide a powerful *in situ* dataset to help improve satellite observations in coastal areas, where accuracy is currently limited [15]. In addition, *in situ* CARICOMP datasets can help ground truth environmental reconstructions of coastal ecosystems based on geochemical analyses of natural archives (e.g. massive corals). Particularly, calibrations of temperature and salinity proxies can be achieved using CARICOMP data. Such calibrations and reconstructions are indispensable to extend time scales prior to monitoring and instrumental records [68–69] and to infer the magnitude of human-induced impacts within the context of natural variability. Because CARICOMP sites are located in areas with contrasting environmental regimes (not only in terms of oceanography but also human influence), the dataset could be used to assess the impact of these potential controls in key physicochemical variables. For example, the CARICOMP data may be useful in identifying and assessing indicators of the long-term effects of marine protected areas (MPA), by comparing sites outside and inside MPAs (e.g. Costa Rica, Colombia, Venezuela: [46,49,70]). Furthermore, CARICOMP data can be used to assess the impact of disturbances. For example, the dataset has been used to show a relationship between high sea surface temperatures and coral bleaching (e.g. [71]). Finally, CARICOMP environmental data may support models of marine ecosystem dynamics in the Caribbean region that facilitate science-based decision-making relating to restoration or conservation management practices.

The CARICOMP program aimed to relate environmental data to observed changes in mangrove, seagrass meadow and coral reef communities over time [22], and this study serves as a first step towards that goal. Long-term changes in seagrass biomass and productivity were reported by van Tussenbroek et al. [72] and the documentation of the changes in mangrove and reef communities are currently under preparation. Large heterogeneity in environmental signals reported here could explain, for example, the variability in responses showed by seagrass meadows in the region [72], a hypothesis that could be tested now that both datasets are available. CARICOMP represented the longest, broadest international effort to manually collect data in coastal ecosystems using standard methodologies. By leveraging efforts of a large group of collaborators from multiple institutions across large spatial scales, CARICOMP’s *in situ* monitoring provides an invaluable source to document the spatial distribution of anthropogenic impacts in the coastal Caribbean. Results from this unparalleled effort highlight the limitations of highly variable coastal *in situ* data, but also the potential for documenting change at regional scales.

## Supporting information

S1 File Site metadata. Word file including metadata for all CARICOMP stations included in the database and mixed effect model fits for temperature and visibility.
(DOCX)

S2 File CARICOMP environmental database. Text file including all CARICOMP’s weekly environmental data.
(TXT)

S3 File Mixed effect models results. Word file including non-linear mixed effect model fits for temperature and visibility.
(DOCX)

## References

[1] Halpern BS, Walbridge S, Selkoe KA, Kappel CV, Micheli F, D’Agrosa C, et al A global map of human impact on marine ecosystems. Science. 2008;319: 948–52. doi: 10.1126/science.1149345 18276889 10.1126/science.1149345

[2] Hoegh-Guldberg O, Mumby PJ, Hooten AJ, Steneck RS, Greenfield P, Gomez E, et al Coral reefs under rapid climate change and ocean acidification. Science. 2007;318: 1737–42. doi: 10.1126/science.1152509 18079392 10.1126/science.1152509

[3] Halpern BS, Longo C, Hardy D, McLeod KL, Samhouri JF, Katona SK, et al An index to assess the health and benefits of the global ocean. Nature. 2012;488: 615–20. doi: 10.1038/nature11397 22895186 10.1038/nature11397

[4] Ehrlich PR, Ehrlich AH. Can a collapse of global civilization be avoided? Proc R Soc Lond B Biol Sci. 2013;280: 20122845. 10.1098/rspb.2012.2845 PMC3574335 23303549

[5] Halpern BS, Frazier M, Potapenko J, Casey KS, Koenig K, Longo C, et al Spatial and temporal changes in cumulative human impacts on the world’s ocean. Nat Commun. 2015;6: 7615 doi: 10.1038/ncomms8615 26172980 10.1038/ncomms8615 PMC4510691

[6] Burke L, Reytar K, Spalding M, Perry A. Reefs at risk revisited. Washington DC: World Resources Institute; 2011 p. 114.

[7] IPCC. Climate change 2013: the physical science basis Contribution of working group I to the fifth assessment report of the Intergovernmental Panel on Climate Change. Cambridge, United Kingdom and New York, NY, USA: Cambridge University Press; 2013 1535 p.

[8] Chollett I, Müller-Karger FE, Heron SF, Skirving W, Mumby PJ. Seasonal and spatial heterogeneity of recent sea surface temperature trends in the Caribbean Sea and southeast Gulf of Mexico. Mar Pollut Bull. 2012;64: 956–65. doi: 10.1016/j.marpolbul.2012.02.016 22406045 10.1016/j.marpolbul.2012.02.016

[9] Cróquer A, Weil E. Spatial variability in distribution and prevalence of Caribbean scleractinian coral and octocoral diseases. II. Genera-level analysis. Dis Aquat Organ. 2009;83: 209–22. doi: 10.3354/dao02012 19402454 10.3354/dao02012

[10] Eakin CM, Morgan JA, Heron SF, Smith TB, Liu G, Alvarez-Filip L, et al Caribbean corals in crisis: record thermal stress, bleaching, and mortality in 2005. PLoS ONE. 2010;5: e13969 doi: 10.1371/journal.pone.0013969 21125021 10.1371/journal.pone.0013969 PMC2981599

[11] Weil E, Cróquer A. Spatial variability in distribution and prevalence of Caribbean scleractinian coral and octocoral diseases. I. Community-level analysis. Dis Aquat Organ. 2009;83: 195–208. doi: 10.3354/dao02011 19402453 10.3354/dao02011

[12] Chen Z, Hu C, Muller-Karger F. Monitoring turbidity in Tampa Bay using MODIS/Aqua 250-m imagery. Remote Sens Environ. 2007;109: 207–20.

[13] Miller RL, Cruise JF. Effects of suspended sediments on coral growth: evidence from remote sensing and hydrologic modeling. Remote Sens Environ. 1995;53: 177–87.

[14] Purkis S, Klemas V, Purkis S, Klemas V. Observing the oceans In: Remote Sensing and Global Environmental Change. John Wiley & Sons Ltd; 2011 p. 204–40.

[15] Hedley J, Roelfsema C, Chollett I, Harborne A, Heron S, Weeks S, et al Remote sensing of coral reefs for monitoring and management: a review. Remote Sens. 2016;8: 118.

[16] Miller RL, Del Castillo CE, McKee BA. Remote sensing of coastal aquatic environments. Springer; 2005.

[17] Barbier EB, Hacker SD, Kennedy C, Koch EW, Stier AC, Silliman BR. The value of estuarine and coastal ecosystem services. Ecol Monogr. 2011;81: 169–93.

[18] Costanza R, d’Arge R, de Groot R, Farber S, Grasso M, Hannon B, et al The value of the world’s ecosystem services and natural capital. Nature. 1997;387: 253–60.

[19] Werdell PJ, Fargion GS, McClain CR, Bailey SW. The SeaWiFS bio-optical archive and storage system (SeaBASS): Current architecture and implementation. Greenbelt: NASA Goddard Space Fight Center; 2002.

[20] Levitus S, Antonov J, Baranova OK, Boyer T, Coleman C, Garcia H, et al The world ocean database. Data Sci J. 2013;12: WDS229–34.

[21] Kjerve B. CARICOMP—Caribbean coral reef, seagrass and mangrove sites Coastal region and small islands papers 3. Paris: UNESCO; 1998 p. 347.

[22] CARICOMP. The Caribbean coastal marine productivity program (CARICOMP). Bull Mar Sci. 2001;69: 819–29.

[23] Koltes KH, Renteria FR, Kjerfve B, Smith SR, Alleng G, Bonair K, et al. Meteorological and oceanographic characterization of coral reef, seagrass and mangrove habitats in the wider Caribbean. In: Proc 8th Int Coral Reef Sym. 1997. p. 651–6.

[24] Sanden P, Hakansson B. Long-term trends in Secchi depth in the Baltic Sea. Limnol Oceanogr. 1996;41: 346–51.

[25] Kuznetsova A, Brockhoff PB, Christensen RHB. lmerTest: Tests in Linear Mixed Effects Models. 2016. https://CRAN.R-project.org/package=lmerTest

[26] Bolker BM, Brooks ME, Clark CJ, Geange SW, Poulsen JR, Stevens MH, White JS. Generalized linear mixed models: a practical guide for ecology and evolution. Trends Ecol Evolut. 2009;24: 127–35. 10.1016/j.tree.2008.10.008 19185386

[27] Weatherhead EC, Reinsel GC, Tiao GC, Meng X-L, Choi D, Cheang W-K, et al Factors affecting the detection of trends: Statistical considerations and applications to environmental data. J Geophys Res Atmospheres. 1998;103: 17149–61.

[28] Pinheiro J, Bates D, DebRoy S, Sarkar D, R Core Team. nlme: Linear and Nonlinear Mixed Effects Models. 2016. http://CRAN.R-project.org/package=nlme

[29] Fabricius KE. Factors determining the resilience of coral reefs to eutrophication: a review and conceptual model In: Dubinsky Z, Stambler N, editors. Coral Reefs: An Ecosystem in Transition. Dordrecht: Springer Netherlands; 2011 p. 493–505.

[30] Lima FP, Wethey DS. Three decades of high-resolution coastal sea surface temperatures reveal more than warming. Nat Commun. 2012;3: 704 doi: 10.1038/ncomms1713 22426225 10.1038/ncomms1713

[31] Hoffman RN, Leidner SM. An introduction to the near—real—time QuikSCAT data. Weather Forecast. 2005;20: 476–93.

[32] Wessel P, Smith WHF. A global, self-consistent, hierarchical, high-resolution shoreline database. J Geophys Res Solid Earth. 1996;101: 8741–8743.

[33] Chollett I, Mumby PJ, Muller-Karger FE, Hu C. Physical environments of the Caribbean Sea. Limnol Oceanogr. 2012;57: 1233–44.

[34] Bivand RS, Pebesma E, Gomez-Rubio V. Applied spatial data analysis with R, Second edition Springer, NY; 2013 http://www.asdar-book.org/

[35] Bivand R, Lewin-Koh N. maptools: Tools for Reading and Handling Spatial Objects. 2016. https://CRAN.R-project.org/package=maptools

[36] Bivand R, Rundel C. rgeos: Interface to Geometry Engine—Open Source (GEOS). 2016. https://CRAN.R-project.org/package=rgeos

[37] Hijmans RJ. raster: Geographic Data Analysis and Modeling. 2015. https://CRAN.R-project.org/package=raster

[38] Chassignet EP, Hurlburt HE, Smedstad OM, Halliwell GR, Hogan PJ, Wallcraft AJ, et al The HYCOM (HYbrid Coordinate Ocean Model) data assimilative system. Mar Environ Monit Predict Pap 36th Int Liège Colloq Ocean Dyn Int Liège Colloq Ocean Dyn. 2007;65: 60–83.

[39] Rainer B. An oceanic general circulation model framed in hybrid isopycnic-Cartesian coordinates. Ocean Model. 2002;4: 55–88.

[40] Balk DL, Deichmann U, Yetman G, Pozzi F, Hay SI, Nelson A. Determining global population distribution: methods, applications and data. In: Simon I. Hay AG and DJ R, editor. Advances in Parasitology. Academic Press; 2006 p. 119–56. 10.1016/S0065-308X(05)62004-0 PMC3154651 16647969

[41] Cinner JE, Huchery C, MacNeil MA, Graham NA, McClanahan TR, Maina J, et al Bright spots among the world’s coral reefs. Nature. 2016;535: 416–9. doi: 10.1038/nature18607 27309809 10.1038/nature18607

[42] Adler RF, Huffman GJ, Chang A, Ferraro R, Xie P-P, Janowiak J, et al The Version-2 Global Precipitation Climatology Project (GPCP) Monthly Precipitation Analysis (1979–Present). J Hydrometeorol. 2003;4: 1147–67.

[43] Huffman GJ, Adler RF, Bolvin DT, Gu G. Improving the global precipitation record: GPCP Version 2.1. Geophys Res Lett. 2009;36: L17808.

[44] Venables WN, Ripley BD. Modern Applied Statistics with S. Fourth New York: Springer; 2002.

[45] Wickham H. ggplot2: Elegant Graphics for Data Analysis. Springer-Verlag New York; 2009.

[46] Cortés J, Fonseca AC, Nivia-Ruiz J, Nielsen-Muñoz V, Samper-Villarreal J, Salas E, et al Monitoring coral reefs, seagrasses and mangroves in Costa Rica (CARICOMP). Rev Biol Trop. 2010;58: 1–22. 21302409

[47] López-Calderón JM, Guzmán HM, Jácome GE, Barnes PA. Decadal increase in seagrass biomass and temperature at the CARICOMP site in Bocas del Toro, Panama. Rev Biol Trop. 2013;61: 1815–1826. 24432536 10.15517/rbt.v61i4.12854

[48] Rodríguez-Martínez RE, Ruíz-Rentería F, van Tussenbroek B, Barba-Santos G, Escalante-Mancera E, Jordán-Garza G, et al Environmental state and tendencies of the Puerto Morelos CARICOMP site, Mexico. Rev Biol Trop. 2010;58: 23–43. 21299094

[49] Rodríguez-Ramírez A, Garzón-Ferreira J, Batista-Morales A, Gil LD, Gómez-López DI, Gómez-Campo K, et al Temporal patterns in coral reef, seagrass and mangrove communities from Chengue bay CARICOMP site (Colombia): 1993–2008. Rev Biol Trop. 2010;58: 45–62. 21299095

[50] Seemann J, González CT, Carballo-Bolaños R, Berry K, Heiss GA, Struck U, et al Assessing the ecological effects of human impacts on coral reefs in Bocas del Toro, Panama. Environ Monit Assess. 2014;186: 1747–1763. doi: 10.1007/s10661-013-3490-y 24254491 10.1007/s10661-013-3490-y

[51] Schluessel P, Emery WJ, Grassl H, Mammen T. On the bulk-skin temperature difference and its impact on satellite remote sensing of sea surface temperature. J Geophys Res Oceans. 1990;95: 13341–13356.

[52] Leichter JJ, Helmuth B, Fischer AM. Variation beneath the surface: quantifying complex thermal environments on coral reefs in the Caribbean, Bahamas and Florida. J Mar Res. 2006; 64: 563–88.

[53] Beaulieu C, Henson SA, Sarmiento JL, Dunne JP, Doney SC, Rykaczewski RR, et al Factors challenging our ability to detect long-term trends in ocean chlorophyll. Biogeosciences, 2013;10: 2711–2724.

[54] Henson SA, Beaulieu C, Lamplitt R. Observing climate change trends in ocean biogeochemistry: when and where. Global Change Biol. 2016;22: 1561–1571. 10.1111/gcb.13152 PMC4785610 26742651

[55] Henson SA, Sarmiento JL, Dunne JP, Bopp L, Lima I, Doney SC, et al Detection of anthropogenic climate change in satellite records of ocean chlorophyll and productivity. Biogeosciences, 2010;7: 621–640.

[56] Weatherhead EC, Stevermer AJ, Schwartz BE. Detecting environmental changes and trends. Phys Chem Earth Parts ABC. 2002;27: 399–403.

[57] Fabricius KE. Effects of terrestrial runoff on the ecology of corals and coral reefs: review and synthesis. Mar Pollut Bull. 2005;50: 125–46. doi: 10.1016/j.marpolbul.2004.11.028 15737355 10.1016/j.marpolbul.2004.11.028

[58] Collin R, D’Croz L, Gondola P, Del Rosario JB. Climate and hydrological factors affecting variation in chlorophyll concentration and water clarity in the Bahia Almirante, Panama. In: Proceedings of the Smithsonian Marine Science Symposium. 2009. p. 323–34.

[59] Taylor MA, Enfield DB, Chen AA. Influence of the tropical Atlantic versus the tropical Pacific on Caribbean rainfall. J Geophys Res Oceans. 2002;107: 3127.

[60] Taylor MA, Whyte FS, Stephenson TS, Campbell JD. Why dry? Investigating the future evolution of the Caribbean Low Level Jet to explain projected Caribbean drying. Int J Climatol. 2013;33: 784–792.

[61] Rauscher SA, Kucharski F, Enfield DB. The role of regional SST warming variations in the drying of Meso-America in future climate projections. J Clim. 2010;24: 2003–2016.

[62] Harvell D, Aronson R, Baron N, Connell J, Dobson A, Ellner S, et al The rising tide of ocean diseases: unsolved problems and research priorities. Front Ecol Environ. 2004;2: 375–82.

[63] Short FT, Wyllie-Echeverria S. Natural and human-induced disturbance of seagrasses. Environ Conserv. 1996; 23: 17–27.

[64] Schaeffer BA, Schaeffer KG, Keith D, Lunetta RS, Conmy R, Gould RW. Barriers to adopting satellite remote sensing for water quality management. Int J Remote Sens. 2013; 34: 7534–7544.

[65] Kennedy EV, Perry CT, Halloran PR, Iglesias-Prieto R, Schonberg CH, Wisshak M, et al Avoiding coral reef functional collapse requires local and global action. Curr Biol. 2013;23: 912–918. doi: 10.1016/j.cub.2013.04.020 23664976 10.1016/j.cub.2013.04.020

[66] Wooldridge SA, Done TJ. Improved water quality can ameliorate effects of climate change on corals. Ecol Appl, 2009; 19: 1492–1499. 19769097 10.1890/08-0963.1

[67] CARICOMP. Caribbean coastal marine productivity (CARICOMP): A research and monitoring network of marine laboratories, parks and reserves. Proc 8th Int Coral Reef Symp. 1997; 1: 641–646.

[68] Grottoli AG, Eakin CM. A review of modern coral d18O and D14C proxy records. Earth Sci Rev. 2007;81: 67–91.

[69] Lough JM. Climate records from corals. Wiley Interdisciplinary Reviews: Climate Change 2010;1: 318–331.

[70] Croquer A, Debrot D, Klein E, Kurten M, Rodríguez S, Bastidas C. What two years of monitoring tell us about Venezuelan coral reefs? The southern Tropical America node of the Global coral reef monitoring network (STA-GCRMN). Rev Biol. Trop. 2010;58: 51–65. 20873040 10.15517/rbt.v58i1.20023

[71] CARICOMP. Studies on Caribbean coral bleaching, 1995–1996. Proc 8th Int Coral Reef Symp. 1997; 1: 673–678.

[72] van Tussenbroek BI, Cortés J, Collin R, Fonseca AC, Gayle PMH, Guzmán HM, et al Caribbean-wide, long-term study of seagrass beds reveals local variations, shifts in community structure and occasional collapse. PLoS ONE. 2014;9: e90600 doi: 10.1371/journal.pone.0090600 24594732 10.1371/journal.pone.0090600 PMC4036797

