# Correction: Widespread local chronic stressors in Caribbean coastal habitats

**DOI:** 10.1371/journal.pone.0192016

**Published:** 2018-01-25

**Authors:** Iliana Chollett, Rachel Collin, Carolina Bastidas, Aldo Cróquer, Peter M. H. Gayle, Eric Jordán-Dahlgren, Karen Koltes, Hazel Oxenford, Alberto Rodriguez-Ramirez, Ernesto Weil, Jahson Alemu, David Bone, Kenneth C. Buchan, Marcia Creary Ford, Edgar Escalante-Mancera, Jaime Garzón-Ferreira, Hector M. Guzmán, Björn Kjerfve, Eduardo Klein, Croy McCoy, Arthur C. Potts, Francisco Ruíz-Rentería, Struan R. Smith, John Tschirky, Jorge Cortés

An affiliation for the 11th author is not indicated. Jahson Alemu is also affiliated with Institute of Marine Affairs, Chaguaramas, Trinidad, Trinidad and Tobago.
