# Supplementary material for: Widespread local chronic stressors in Caribbean coastal habitats
Source: PLoS One. 2017 Dec 20;12(12):e0188564. doi: 10.1371/journal.pone.0188564 (PMC5737976; doi:10.1371/journal.pone.0188564)
Supplement: S1 File — Word file including metadata for all CARICOMP stations included in the database and mixed effect model fits for temperature and visibility. (DOCX) [file pone.0188564.s001.docx]

The following supplement accompanies the article

**Evidence for widespread local chronic stressors in Caribbean coastal habitats**

*Iliana Chollett^*^, Rachel Collin, Carolina Bastidas, Aldo Cróquer, Peter MH Gayle, Eric Jordán-Dahlgren, Karen Koltes, Hazel Oxenford, Alberto Rodríguez-Ramírez, Ernesto Weil, Jahson Alemu, David Bone, Kenneth C Buchan, Marcia Creary Ford, Edgar Escalante-Mancera, Jaime Garzón-Ferreira, Hector M Guzmán, Björn Kjerfve, Eduardo Klein, Croy McCoy, Arthur C Potts, Francisco Ruíz-Rentería, Struan R Smith, John Tschirky and Jorge Cortés*

*Corresponding author Iliana.chollett@gmail.com

Metadata for all CARICOMP stations included in the database can be found below. The following two tables provide metadata to understand the CARICOMP dataset included in S2 File, which contains a tab-delimited text file with all the weekly information gathered by the CARICOMP network up to 2015. If using this dataset for research, please acknowledge its source by citing this publication.

**Table A.** Metadata for all CARICOMP stations included in the database (Supplementary Material 2), which can be cross-referenced by the fields ‘site’ and ‘ecosystem’

| Country | Acronym | Site | Ecosystem | Latitude | Longitude |
| --- | --- | --- | --- | --- | --- |
| Bahamas | BAH | San Salvador | Coral Reef | 24.0367 | -74.5325 |
| Bahamas | BAH | San Salvador | Seagrass Beds | 23.9504 | -74.5417 |
| Barbados | BAR | Bellairs | Coral Reef | 13.1917 | -59.6418 |
| Barbados | BAR | Bellairs | Mangrove | 13.0717 | -59.5769 |
| Barbados | BAR | Bellairs | Seagrass Beds | 13.0681 | -59.5783 |
| Belize | BEL | Carrie Bow Cay | Coral Reef | 16.8000 | -88.0667 |
| Belize | BEL | Carrie Bow Cay | Mangrove | 16.8333 | -88.1167 |
| Belize | BEL | Carrie Bow Cay | Seagrass Beds | 16.8253 | -88.0993 |
| Bermuda | BER | Hog Breaker Reef | Coral Reef | 32.3439 | -64.8648 |
| Bermuda | BER | Hungry Bay | Mangrove | 32.2883 | -64.7583 |
| Bermuda | BER | North Seagrass | Seagrass Beds | 32.4007 | -64.7985 |
| Bermuda | BER | West Seagrass | Seagrass Beds | 32.4000 | 64.8000 |
| Bonaire, N.A. | BON | Barcadera Reef | Coral Reef | 12.1954 | -68.3014 |
| Colombia | COL | Chengue Bay | Coral Reef | 11.3278 | -74.1285 |
| Colombia | COL | Chengue Bay | Mangrove | 11.3166 | -74.1281 |
| Colombia | COL | Chengue Bay | Seagrass Beds | 11.3213 | -74.1266 |
| Colombia | COL2 | Cotton Cay North | Seagrass Beds | 12.5596 | -81.6996 |
| Colombia | COL2 | La Iguana 2B | Coral Reef | 12.5013 | -81.7340 |
| Colombia | COL2 | San Andres | Seagrass Beds | 12.5833 | -81.7000 |
| Colombia | COL2 | Wild Life 1B | Coral Reef | 12.5162 | -81.7320 |
| Costa Rica | CRI | Rio Perezoso | Coral Reef | 9.7306 | -82.8089 |
| Costa Rica | CRI | Rio Perezoso | Seagrass Beds | 9.7370 | -82.8067 |
| Cuba | CUB | Cayo Coco A | Coral Reef | 22.5636 | -78.4386 |
| Cuba | CUB | Cayo Coco B | Coral Reef | 22.5633 | -78.4450 |
| Curaçao | CUR | Spaanse Water | Coral Reef | 12.0603 | -68.8575 |
| Curaçao | CUR | Spaanse Water | Mangrove | Unknown | Unknown |
| Curaçao | CUR | Spaanse Water | Seagrass Beds | 12.0833 | -68.8667 |
| Dominican Republic | DRE | Parque Nacional del Este | Seagrass Beds | 18.2255 | -68.7683 |
| Jamaica | JAM | Discovery Bay | Coral Reef | 18.4722 | -77.4136 |
| Jamaica | JAM | Discovery Bay | Mangrove | 18.4692 | -77.4154 |
| Jamaica | JAM | Discovery Bay | Seagrass Beds | 18.4711 | -77.4135 |
| Mexico | MEX | Puerto Morelos | Coral Reef | 20.8777 | -86.8451 |
| Mexico | MEX | Puerto Morelos | Seagrass Beds | 20.8677 | -86.8671 |
| Panama | PAN | STRI_colo | Coral Reef | 9.3485 | -82.2660 |
| Panama | PAN | STRI_colo | Mangrove | 9.3521 | -82.2590 |
| Panama | PAN | STRI_colo | Seagrass Beds | 9.3517 | -82.2578 |
| Puerto Rico | PUR | La Parguera | Coral Reef | 17.9349 | -67.0485 |
| Puerto Rico | PUR | La Parguera | Seagrass Beds | 17.9549 | -67.0435 |
| Saba, N.A. | SAB | Ladder Labyrinth | Coral Reef | 17.6261 | -63.2599 |
| Trinidad and Tobago | TAT | Buccoo Reef | Coral Reef | 11.1894 | -60.8411 |
| Trinidad and Tobago | TAT | Buccoo Reef | Seagrass Beds | Unknown | Unknown |
| USA | USA | Long Key | Seagrass Beds | 24.8000 | -80.7167 |
| Venezuela | VEN | Caiman | Coral Reef | 10.8517 | -68.2322 |
| Venezuela | VEN | Cayo Sombrero | Coral Reef | 10.8833 | -68.2125 |
| Venezuela | VEN | Parque Nacional Morrocoy | Mangrove | 10.8358 | -68.2608 |
| Venezuela | VEN | Parque Nacional Morrocoy | Seagrass Beds | 10.8581 | -68.2909 |
| Venezuela | VEN2 | Punta Ballena 1 | Coral Reef | 10.9981 | -63.7700 |
| Venezuela | VEN2 | Punta de Mangle | Mangrove | 10.8636 | -64.0575 |
| Venezuela | VEN2 | Punta de Mangle | Seagrass Beds | 10.8636 | -64.0575 |

**Table B.** Description of fields included in the database (Supplementary Material 2)

| **Field name** | **Field type** | **Description** |
| --- | --- | --- |
| Site | Text | Site name |
| Date | Numeric, integer | Date in which record was taken, in windows Excel numeric format |
| Ecosystem | Text | Name of coastal ecosystem, either “Coral Reef”, “Seagrass bed” or “Mangrove” |
| Temp | Numeric | Sea water temperature at 0.5 m deep in °C. No data=NA |
| Sal | Numeric | Salinity at 0.5 m deep in psu. No data=NA |
| Secchi | Numeric | Secchi disk depth (m) at which the disk ceases to be visible. No data=NA |
|  |  |  |
