# Supplementary material for: Widespread local chronic stressors in Caribbean coastal habitats
Source: PLoS One. 2017 Dec 20;12(12):e0188564. doi: 10.1371/journal.pone.0188564 (PMC5737976; doi:10.1371/journal.pone.0188564)
Supplement: S3 File — Word file including non-linear mixed effect model fits for temperature and visibility. (DOCX) [file pone.0188564.s003.docx]

The following supplement accompanies the article

**Evidence for widespread local chronic stressors in Caribbean coastal habitats**

*Iliana Chollett^*^, Rachel Collin, Carolina Bastidas, Aldo Cróquer, Peter MH Gayle, Eric Jordán-Dahlgren, Karen Koltes, Hazel Oxenford, Alberto Rodríguez-Ramírez, Ernesto Weil, Jahson Alemu, David Bone, Kenneth C Buchan, Marcia Creary Ford, Edgar Escalante-Mancera, Jaime Garzón-Ferreira, Hector M Guzmán, Björn Kjerfve, Eduardo Klein, Croy McCoy, Arthur C Potts, Francisco Ruíz-Rentería, Struan R Smith, John Tschirky and Jorge Cortés*

*Corresponding author Iliana.chollett@gmail.com

Mixed effect model fits for temperature and visibility can be found below.

**Table A.** Non-linear mixed effect model fit (gnls) for temperature data. Site, ecosystem, station acronym, number of years with data (Years), trend (°C year^-1^), p-value, and site-specific temporal autocorrelation parameter (φ), model residuals and number of years needed to detect a trend of 0.05°C year^-1^. Significant results (p<0.05) in bold.

| **Site** | **Ecosystem** | **Years** | **Trend** | **p-value** | **φ** | **Residuals** | **Years needed** |
| --- | --- | --- | --- | --- | --- | --- | --- |
| **Barcadera Reef** | **Coral Reef** | **4** | **-0.33** | **0.04** | **0.64** | **0.43** | **18.28** |
| Bellairs | Coral Reef | 7 | -0.01 | 0.81 | 0.66 | 0.47 | 20.21 |
| Bellairs | Seagrass Beds | 5 | 0.15 | 0.28 | 0.25 | 0.55 | 13.16 |
| Caiman | Coral Reef | 8 | 0.05 | 0.30 | -0.17 | 0.84 | 13.12 |
| Carrie Bow Cay | Coral Reef | 22 | -0.01 | 0.42 | 0.34 | 0.61 | 15.56 |
| Carrie Bow Cay | Seagrass Beds | 22 | -0.01 | 0.40 | 0.33 | 0.88 | 19.51 |
| **Chengue Bay** | **Coral Reef** | **20** | **0.05** | **0.00** | **0.31** | **0.75** | **17.27** |
| Chengue Bay | Mangrove | 20 | 0.01 | 0.53 | 0.24 | 0.85 | 17.61 |
| **Chengue Bay** | **Seagrass Beds** | **20** | **0.04** | **0.00** | **0.24** | **0.71** | **15.61** |
| Discovery Bay | Coral Reef | 11 | -0.01 | 0.75 | 0.46 | 0.49 | 15.21 |
| Discovery Bay | Mangrove | 11 | 0.01 | 0.45 | 0.26 | 0.29 | 8.77 |
| Discovery Bay | Seagrass Beds | 11 | -0.04 | 0.21 | 0.44 | 0.53 | 15.75 |
| Hog Breaker Reef | Coral Reef | 10 | 0.05 | 0.39 | 0.30 | 1.04 | 21.28 |
| La Parguera | Coral Reef | 22 | 0.02 | 0.09 | 0.50 | 0.53 | 16.86 |
| La Parguera | Seagrass Beds | 22 | 0.01 | 0.25 | 0.54 | 0.60 | 19.47 |
| Ladder Labyrinth | Coral Reef | 6 | -0.10 | 0.19 | 0.49 | 0.32 | 11.86 |
| Long Key | Seagrass Beds | 9 | 0.07 | 0.69 | -0.02 | 1.41 | 20.21 |
| **North Seagrass** | **Seagrass Beds** | **10** | **0.11** | **0.00** | **0.17** | **0.82** | **16.18** |
| **P.N.Morrocoy** | **Coral Reef** | **13** | **0.13** | **0.00** | **0.36** | **0.95** | **21.25** |
| P. N. Morrocoy | Mangrove | 20 | 0.00 | 0.97 | 0.54 | 1.31 | 32.69 |
| P. N. Morrocoy | Seagrass Beds | 21 | 0.03 | 0.42 | 0.61 | 1.46 | 39.57 |
| Puerto Morelos | Coral Reef | 14 | 0.00 | 0.76 | 0.24 | 0.50 | 12.26 |
| Puerto Morelos | Seagrass Beds | 14 | -0.01 | 0.55 | -0.13 | 0.58 | 10.50 |
| Punta de Mangle | Mangrove | 10 | -0.03 | 0.31 | 0.13 | 0.70 | 14.24 |
| Punta de Mangle | Seagrass Beds | 10 | 0.01 | 0.61 | 0.14 | 0.71 | 14.38 |
| STRI_colo | Coral Reef | 17 | 0.02 | 0.12 | 0.45 | 0.51 | 15.62 |
| **STRI_colo** | **Mangrove** | **17** | **0.03** | **0.05** | **0.45** | **0.55** | **16.28** |
| STRI_colo | Seagrass Beds | 17 | 0.02 | 0.19 | 0.40 | 0.56 | 15.53 |

|  |  |  |  |  |  |  |
| --- | --- | --- | --- | --- | --- | --- |
|  |  |  |  |  |  |  |
|  |  |  |  |  |  |  |
|  |  |  |  |  |  |  |
|  |  |  |  |  |  |  |
|  |  |  |  |  |  |  |
|  |  |  |  |  |  |  |
|  |  |  |  |  |  |  |
|  |  |  |  |  |  |  |
|  |  |  |  |  |  |  |
|  |  |  |  |  |  |  |
|  |  |  |  |  |  |  |
|  |  |  |  |  |  |  |
|  |  |  |  |  |  |  |
|  |  |  |  |  |  |  |
|  |  |  |  |  |  |  |
|  |  |  |  |  |  |  |
|  |  |  |  |  |  |  |
|  |  |  |  |  |  |  |
|  |  |  |  |  |  |  |
|  |  |  |  |  |  |  |
|  |  |  |  |  |  |  |
|  |  |  |  |  |  |  |
|  |  |  |  |  |  |  |
|  |  |  |  |  |  |  |
|  |  |  |  |  |  |  |
|  |  |  |  |  |  |  |
|  |  |  |  |  |  |  |
|  |  |  |  |  |  |  |

**Table B.** Mixed-effect model fit (gls) for visibility data. Site, ecosystems, number of years with data (Years), trend (m year^-1^), p-value, and site-specific temporal autocorrelation parameter (φ), model residuals and number of years needed to detect a trend of 0.05°m year^-1^. Significant results (p<0.05) in bold.

| **Site** | **Ecosystem** | **Years** | **Trend** | **p-value** | **φ** | **Residuals** | **Years needed** |
| --- | --- | --- | --- | --- | --- | --- | --- |
| Barcadera Reef | Coral Reef | 4 | 0.07 | 0.17 | 0.59 | 1.92 | 45.69 |
| Bellairs | Coral Reef | 7 | 0.19 | 0.14 | 0.90 | 5.81 | 247.22 |
| **Bellairs** | **Seagrass Beds** | **5** | **-0.10** | **<0.005** | **0.21** | **1.47** | **24.72** |
| Caiman | Coral Reef | 8 | 0.01 | 0.50 | 0.34 | 2.71 | 41.83 |
| **Carrie Bow Cay** | **Coral Reef** | **22** | **-0.02** | **<0.005** | **0.36** | **4.01** | **55.50** |
| **Carrie Bow Cay** | **Seagrass Beds** | **22** | **-0.01** | **<0.005** | **0.37** | **2.07** | **35.87** |
| **Chengue Bay** | **Coral Reef** | **20** | **-0.01** | **0.01** | **0.10** | **2.41** | **31.64** |
| Chengue Bay | Seagrass Beds | 20 | 0.00 | 0.12 | 0.22 | 1.73 | 27.78 |
| **Discovery Bay** | **Coral Reef** | **11** | **-0.07** | **<0.005** | **0.51** | **4.05** | **66.41** |
| **Discovery Bay** | **Seagrass Beds** | **11** | **0.05** | **<0.005** | **0.48** | **3.44** | **57.25** |
| **Hog Breaker Reef** | **Coral Reef** | **10** | **0.11** | **0.02** | **0.37** | **10.63** | **107.79** |
| **La Parguera** | **Coral Reef** | **22** | **-0.02** | **<0.005** | **0.39** | **2.74** | **44.66** |
| **La Parguera** | **Seagrass Beds** | **22** | **-0.01** | **<0.005** | **0.26** | **1.66** | **27.86** |
| **Ladder Labyrinth** | **Coral Reef** | **6** | **-0.09** | **<0.005** | **0.03** | **2.90** | **33.87** |
| Long Key | Seagrass Beds | 9 | 0.01 | 0.27 | 0.09 | 0.71 | 13.76 |
| North Seagrass | Seagrass Beds | 10 | 0.01 | 0.49 | 0.32 | 3.05 | 44.65 |
| P.N.Morrocoy | Coral Reef | 13 | 0.00 | 0.87 | 0.07 | 3.98 | 43.20 |
| **P.N. Morrocoy** | **Mangrove** | **20** | **0.00** | **0.04** | **-0.09** | **1.22** | **17.62** |
| P.N. Morrocoy | Seagrass Beds | 21 | 0.00 | 0.91 | 0.25 | 2.31 | 34.70 |
| Puerto Morelos | Coral Reef | 14 | 0.00 | 0.95 | -0.03 | 2.55 | 29.85 |
| Puerto Morelos | Seagrass Beds | 14 | 0.00 | 0.89 | 0.40 | 2.66 | 44.29 |
| **Punta de Mangle** | **Seagrass Beds** | **10** | **-0.01** | **<0.005** | **0.40** | **0.93** | **21.90** |
| Rio Perezoso | Seagrass Beds | 10 | 0.00 | 0.55 | 0.19 | 1.95 | 29.27 |
| **STRI_colo** | **Coral Reef** | **17** | **-0.01** | **<0.005** | **0.18** | **2.18** | **31.46** |
| STRI_colo | Seagrass Beds | 17 | 0.01 | 0.10 | 0.39 | 2.55 | 42.29 |
